# Regulation of TGF-*β* Signal Transduction

**DOI:** 10.1155/2014/874065

**Published:** 2014-09-23

**Authors:** Bing Zhao, Ye-Guang Chen

**Affiliations:** The State Key Laboratory of Biomembrane and Membrane Biotechnology, Tsinghua-Peking Center for Life Sciences, School of Life Sciences, Tsinghua University, Beijing 100084, China

## Abstract

Transforming growth factor-*β* (TGF-*β*) signaling regulates diverse cellular processes, including cell proliferation, differentiation, apoptosis, cell plasticity, and migration. TGF-*β* signaling can be mediated by Smad proteins or other signaling proteins such as MAP kinases and Akt. TGF-*β* signaling is tightly regulated at different levels along the pathways to ensure its proper physiological functions in different cells and tissues. Deregulation of TGF-*β* signaling has been associated with various kinds of diseases, such as cancer and tissue fibrosis. This paper focuses on our recent work on regulation of TGF-*β* signaling.

## 1. Introduction

Transforming growth factor-*β* (TGF-*β*) family is a group of structurally related growth factors, which includes TGF-*β*, activin, nodal, bone morphogenetic proteins (BMPs), and others. These growth factors play critical roles in regulating a wide range of biological processes during embryonic development and adult tissue homeostasis, and deregulation of the signal transduction has been associated with many human diseases, including cancer and tissue fibrosis [1–3]. TGF-*β* signaling is initiated by the binding of TGF-*β* to its serine and threonine kinase receptors, the type II and type I receptors on the cell membrane. Ligand binding triggers the formation of the receptor heterocomplex, in which type II receptor phosphorylates type I receptor at the threonine and serine residues in its TTSGSGSG motif, leading to the activation of type I receptor [1, 4, 5]. The activated type I receptor recruits and phosphorylates the R-Smad proteins, which then form a heterocomplex with the co-Smad Smad4. The Smad complexes are then accumulated in the nucleus and regulate transcription of the target genes by cooperating with other cofactors [6, 7].

For each member of the TGF-*β* family, they have their own combination of type I and type II receptors and R-Smads. For TGF-*β* signaling, the type I receptor T*β*RI/ALK5 and the type II receptor T*β*RII are employed to activate Smad2/3. For BMP signaling, ALK1/2/3/6 can activate Smad1/5/8 with type II receptor BMPRII, ActRII, and ActRIIB [8, 9]. ALK4/7 can activate Smad2/3 with ActRII and ActRIIB to mediate activin/nodal signaling [10, 11]. In addition, TGF-*β* can also activate mitogen-activating protein kinases (MAPKs) including ERK, p38 and JNK, phosphatidylinositol 3 kinase (PI3K)/Akt, and small GTPases [12–14]. In this review, we mainly summarize our work on the regulation of the activity and stability of TGF-*β* receptors and Smads, highlighting the current understanding and perspectives of TGF-*β* signaling modulation.

## 2. Membrane Trafficking Regulates the Activity and Stability of TGF-***β*** Receptors

Cell surface receptors are internalized through two major endocytic pathways: clathrin-mediated endocytosis and lipid raft/caveolae-mediated endocytosis [15–17]. Clathrin-mediated endocytosis is the best characterized pathway, which is employed by many cell surface receptors such as G protein-coupled receptors, tyrosine kinase receptors, low-density lipoprotein receptor, and transferring receptor [18]. The receptors are first concentrated on the clathrin-coated pits, which are assembled on the cytoplasmic face of the plasma membrane by the recruitment of the adaptor complex AP2, clathrin, and other accessory proteins such as Eps15, epsin, disabled-2, synaptotagmin, and amphiphysin [19–21]. These pits undergo invagination and then pinch off from the plasma membrane in a dynamin GTPase-dependent manner [22]. After uncoating with dissociation of adaptors and clathrin, the vesicle is fused with early endosomes.

Besides clathrin-coated pits, cholesterol-enriched, and specialized detergent-insoluble lipid rafts can also be found in the plasma membrane, which can serve as signaling centers for nitric oxide, calcium, G protein-coupled receptors, and protein tyrosine kinases, or as virus entrance [23, 24]. Some of these membrane microdomains are specialized as caveolae in the presence of caveolin. Caveolae mediates the internalization of various proteins such as choleratoxin, glycosylphosphatidylinositol (GPI)-anchored proteins, endothelin receptor, and growth hormone receptor [25, 26]. The internalized cargos are transported to not well-characterized caveosomes and eventually to later endosomes or lysosomes.

**Figure 1:**
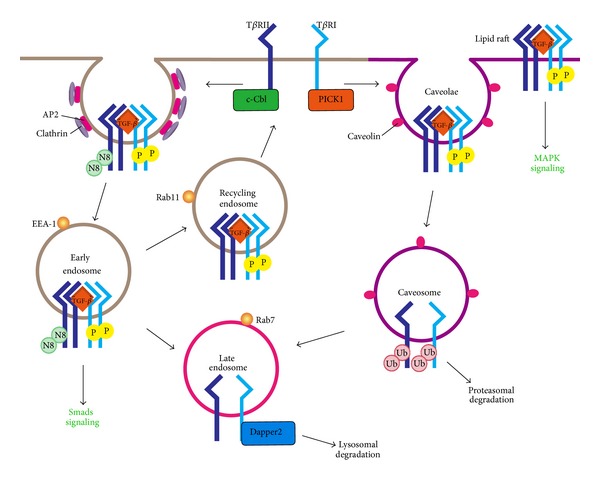
Membrane trafficking regulates the activity and stability of TGF-*β* receptors. Internalization of TGF-*β* receptors through clathrin-dependent endocytosis enhances TGF-*β*-Smad signaling, whereas caveolin-mediated endocytosis promotes the ubiquitination and degradation of the receptors and thus the turnoff of signaling. c-Cbl neddylates T*β*RII and facilitates its clathrin-dependent endocytosis, while PICK1 promotes lipid raft/caveolae localization and caveolin-mediated endocytosis of T*β*RI. Dapper2 locates in late endosomes and accelerates the lysosomal degradation of T*β*RI. The lipid raft localization of TGF-*β* receptors is critical for TGF-*β*-mediated MAPK activation.

TGF-*β* receptors are partitioned between the lipid rafts and nonraft areas on the plasma membrane [27–32]. Ligand binding to its receptor at the cell surface not only initiates signaling events but also triggers internalization of both ligand and receptors. We and others have demonstrated that TGF-*β* receptors can be endocytosed via clathrin-coated vesicles as TGF-*β* endocytosis can be blocked by potassium depletion and the GTPase deficient dynamin K44A mutant [33–35]. Internalization of TGF-*β* receptors through clathrin-dependent endocytosis to EEA1-positive endosomes is more likely to promote signaling as the FYVE domain-containing protein SARA are enriched in EEA1-positive endosomes and can facilitate R-Smads activation [36–38]. To support this idea, we found that endofin, which share a homology with SARA, can interact with TGF-*β* receptors and Smad4 and promote TGF-*β*-induced Smad complex formation [39]. The internalized receptors can be recycled to the membrane in a Rab11-dependent manner [40]. TGF-*β* receptors located in lipid raft regions enter cells via lipid raft/caveolae and are found in caveolin-positive vesicles [36]. Lipid raft/caveolae is indicated to facilitate the degradation of TGF-*β* receptors and therefore turnoff of TGF-*β* signaling (Figure 1).

The partitioning and internalization of TGF-*β* receptors are regulated processes [41]. One of the major regulators we identified is Casitas B-lineage lymphoma (*c-Cbl*), a protooncogene with widespread mutations in hematopoietic malignancies [42]. Unlike its classic role as a ubiquitin E3 ligase mediating receptor tyrosine kinases (RTKs) ubiquitination and degradation, c-Cbl interacts with T*β*RII and conjugates neural precursor cell-expressed, developmentally downregulated 8 (NEDD8), a ubiquitin-like protein, to T*β*RII at Lys556 and Lys567 [43]. Neddylation has been reported to regulate substrate protein activity, stability, and subcellular localization [44]. In the case of T*β*RII, we demonstrated that c-Cbl-mediated neddylation could target T*β*RII into EEA1-positive early endosomes and prevent its endocytosis to caveolin-positive compartments. Consequently, c-Cbl stabilizes T*β*RII by attenuating its ubiquitination and degradation and thereby enhances cellular TGF-*β* responsiveness.

It has been well established that c-Cbl mutations contribute to leukemia by negatively regulating the activity and stability of receptor tyrosine kinases [45–47]. Besides, disruption of TGF-*β* signaling, which is a major antiproliferation and prodifferentiation signal for hematopoietic stem/progenitor cells [48], greatly promotes lymphoblastic and myeloid leukemia in mouse models [49, 50]. We demonstrated that c-Cbl overexpression stabilizes T*β*RII and sensitizes leukemia cells to TGF-*β*-induced growth inhibition. We also identified a neddylation-activity-defective c-Cbl mutation from leukemia patients, implying that c-Cbl inactivation contributes to leukemia development not only by amplifying the mitogenic signals from RTKs, but also by releasing the antiproliferative effects of TGF-*β*.

We demonstrated that PICK1 (protein that interacts with C kinase 1), opposite to c-Cbl, promotes lipid raft/caveolae localization and caveolin-mediated endocytosis of TGF-*β* receptors [51]. As an adaptor protein, PICK1 has been shown to interact with a number of membrane proteins and regulate their subcellular trafficking, such as AMPAR [52–55], acid-sensing ion channel [56], and ErbB2/Her-2 [57]. Our biochemical analyses reveal that PICK1 directly interacts with the C-terminus of T*β*RI via its PDZ domain and acts as a scaffold protein to enhance the interaction between T*β*RI and caveolin-1, leading to increased lipid raft/caveolae localization [51]. Therefore, PICK1 increases caveolin-mediated endocytosis, ubiquitination, and degradation of T*β*RI and suppresses TGF-*β* signaling.

Previous studies associated the deviant expression of PICK1 in brain with mental disorders such as schizophrenia [58–60]. However, PICK1 is ubiquitously expressed in many organs outside the nervous system, and its physiological functions have not been fully investigated. By modulating the signaling, PICK1 may participate in TGF-*β*-related processes. Indeed, we observed a significant negative correlation between PICK1 expression and T*β*RI or phospho-Smad2 levels in human breast tumors, indicating that PICK1 may be involved in breast cancer development through inhibition of TGF-*β* signaling [51]. This idea is also supported by other reports suggesting that PICK1 is associated with human cancer development [57, 61–63].

In fact, distribution of TGF-*β* receptors in lipid rafts does not simply promote receptor degradation. We showed that localization of TGF-*β* receptors in the lipid raft regions is required for TGF-*β*-mediated MAPK activation. Disturbance of distribution of TGF-*β* receptors in lipid rafts by cholesterol depletion blocks TGF-*β*-induced MAPK activation and epithelial-mesenchymal transition (EMT) [64]. Consistent with this, specific targeting of the intracellular domain of T*β*RI to lipid rafts directly activates ERK and triggers EMT. This suggests a distinct role of lipid rafts in controlling the canonical TGF-*β*/Smad signaling and the TGF-*β*/noncanonical MAPK signaling.

We have also identified another regulator of TGF-*β* receptors trafficking and turnover, Dapper2. Interacting with Dishevelled with its C-terminal PDZ-binding motif, Dapper1 was first identified as a Wnt signaling antagonist in* Xenopus* [65]. Then, the inhibitory effect of Dapper2 on TGF-*β*/nodal signaling was demonstrated in zebrafish mesoderm induction [66], and its function is later found to be conserved in mammalian cells [67]. Dapper2 preferentially interacts with T*β*RI/ALK5 and activin receptor ActRIB/ALK4 in the Rab7-positive late endosomes and accelerates their lysosomal degradation, suggesting that Dapper2 facilitates the transport of endocytosed receptors from late endosomes to lysosomes. However, its detailed mechanism is unclear.

## 3. Regulation of TGF-***β*** Receptor Ubiquitination and Stability

**Figure 2:**
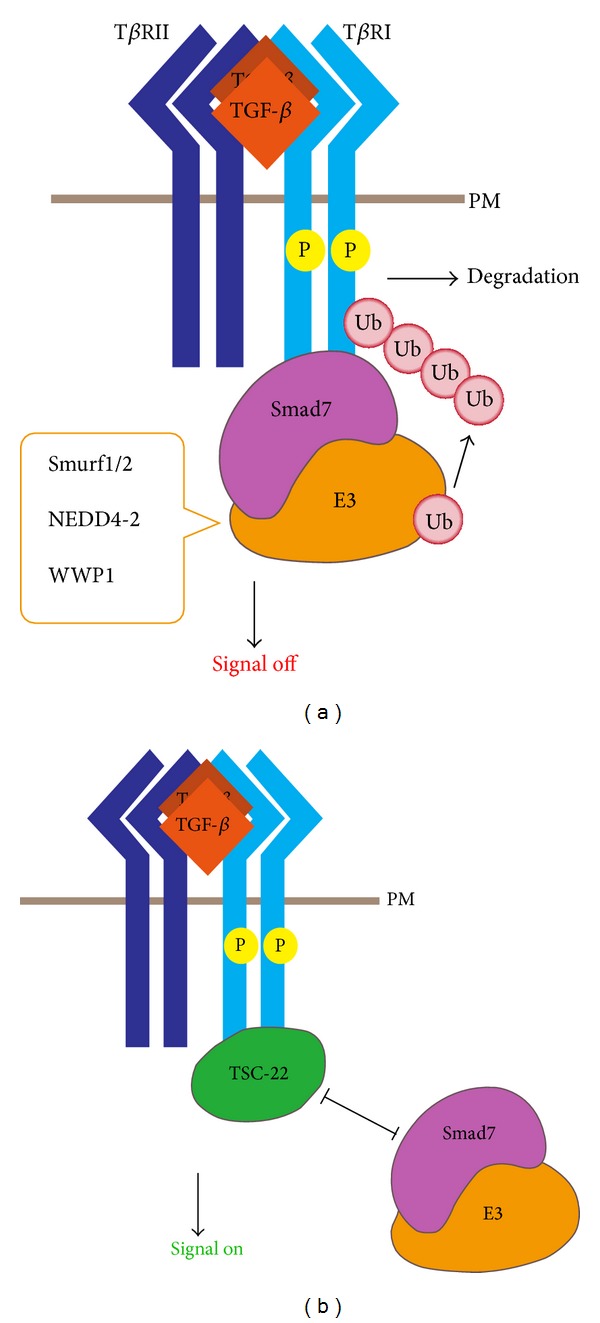
Regulation of TGF-*β* receptor degradation and expression. (a) TGF-*β* receptors localized in lipid raft/caveolae and caveolin-1-positive vesicles undergo ubiquitination. Smad7 recruits HECT domain-containing E3 ligases to mediate ubiquitination and degradation of T*β*RI. (b) TSC-22 competes with Smad7/Smurfs for T*β*RI binding and therefore decreases the ubiquitination and degradation of the receptor, leading to enhanced TGF-*β* signaling. PM: plasma membrane.

TGF-*β* receptors localized in lipid raft/caveolae and caveolin-1-positive vesicles undergo ubiquitination-mediated degradation [36, 68, 69]. Recruitment of the WW-HECT-type E3 ubiquitin ligases Smurf1, Smurf2, NEDD4-2 and WWP1 to T*β*RI is essential for its ubiquitination, in which process Smad7 acts as a critical adaptor [70]. Smad7 can bind to T*β*RI and HECT domain-containing E3 ligases and thus facilitate the assembly of the T*β*RI-Smad7-E3 complex, in which both T*β*RI and Smad7 are ubiquitinated and degradated [71–75] (Figure 2(a)).

T*β*RI ubiquitination is finely controlled by multiple proteins, one of which we found is TGF-*β*-stimulated clone 22 (TSC-22). TSC-22, which was first reported as a TGF-*β*-upregulated gene in MC3T3E1 mouse osteoblastic cells, contains a leucine zipper-like structure and a nuclear export signal [76]. Accumulated evidence indicates that TSC-22 has an antiproliferative activity and is downregulated in several types of tumor cells [77–82]. We identified TSC-22 as a T*β*RI-binding partner using a yeast two-hybrid screen [83]. As a TGF-*β* target, TSC-22 can disrupt the binding of Smad7/Smurfs with T*β*RI and therefore decrease the ubiquitination and degradation of the receptor, leading to enhanced TGF-*β* signaling [83] (Figure 2(b)). This positive-feedback loop may be involved in myocardial fibrosis as an elevated TSC-22 level was correlated with TGF-*β* signaling activation and enhanced expression of fibrotic genes in the isoproterenol-induced heart fibrosis model. However, it is unclear whether TSC-22 prevents Smad7-induced receptor ubiquitination/degradation in lipid rafts or in nonraft regions.

## 4. Regulation of TGF-***β*** Receptor Expression

Although modulation of receptor activities is a critical step for TGF-*β* signaling regulation, the regulation of TGF-*β* receptor expression is also important. Histone acetylation has been indicated to regulate TGF-*β* receptor expression [84–87]. Other mechanisms may be also employed to control their transcription. In search for miRNAs interfering type I receptor expression, we found that microRNA miR-24 reduces the mRNA and protein levels of human activin type I receptor ALK4 (ALK4) by targeting the 3′-untranslated region of ALK4 mRNA and inhibits activin signaling [88]. Consequently, miR-24 represses the activin-mediated erythroid differentiation of K562 cells, erythroid colony formation, and maturation of human CD34+ hematopoietic progenitor cells. T*β*RII expression is also repressed by mir-106b [89].

## 5. Modulation of Smad Activation

**Figure 3:**
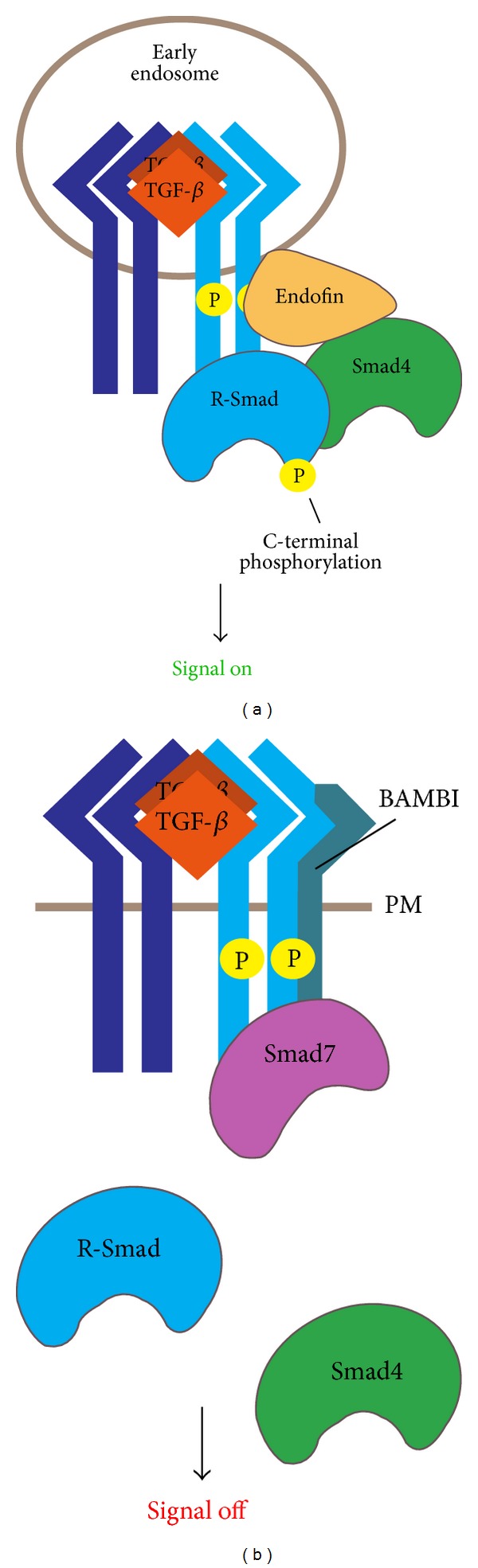
Modulation of Smad activation. (a) Activated T*β*RI recruits and phosphorylates Smad2/3 at the C-terminal, and then the phosphorylated Smad2/3 binds Smad4 to form a Smad heterocomplex to mediate signal transduction. Endofin recruits Smad4 to the receptor complex in early endosomes and facilitates the association of receptor-activated Smad2/3 with Smad4. (b) BAMBI forms a ternary complex with receptors and Smad7 and inhibits the interaction between T*β*RI and Smad3, impairing Smad3 activation. PM: plasma membrane.

Upon being phosphorylated by T*β*RII, the activated T*β*RI recruits and phosphorylates Smad2/3 at the C-terminal (Figure 3(a)). Various proteins associated with the receptors complex have been reported to regulate R-Smad recruitment [90], such as SARA and endofin as mentioned above. BMP and activin membrane-bound inhibitor (BAMBI) has been reported as a general antagonist of TGF-*β* family members. Acting as a pseudoreceptor, BAMBI interferes with the interaction between type I and type II receptors of the TGF-*β* family [91]. In addition to blocking the heterocomplex formation of TGF-*β* receptors, our recent work showed that BAMBI cooperates with Smad7 to inhibit TGF-*β* signaling [92]. BAMBI can form a ternary complex with Smad7 and T*β*RI and inhibit the interaction between T*β*RI and Smad3, which impairs Smad3 activation (Figure 3(b)). Besides, we also found that p21-activated kinase 2 (PAK2) can directly phosphorylate Smad2 at Ser417, which interferes with the T*β*RI-Smad2 association and thus blocks TGF-*β*-induced Smad2 activation and signaling [93].

Phosphorylated Smad2/3 binds Smad4 to form a Smad heterocomplex, which mediates downstream signal transduction. We have reported that the FYVE domain-containing protein endofin can interact with both T*β*RI and Smad4 [39]. As a scaffold protein, endofin recruits Smad4 to T*β*RI in early endosomes and facilitates the association of receptor-activated Smad2 with Smad4 (Figure 3(a)).

## 6. Regulation of Smad Activity

**Figure 4:**
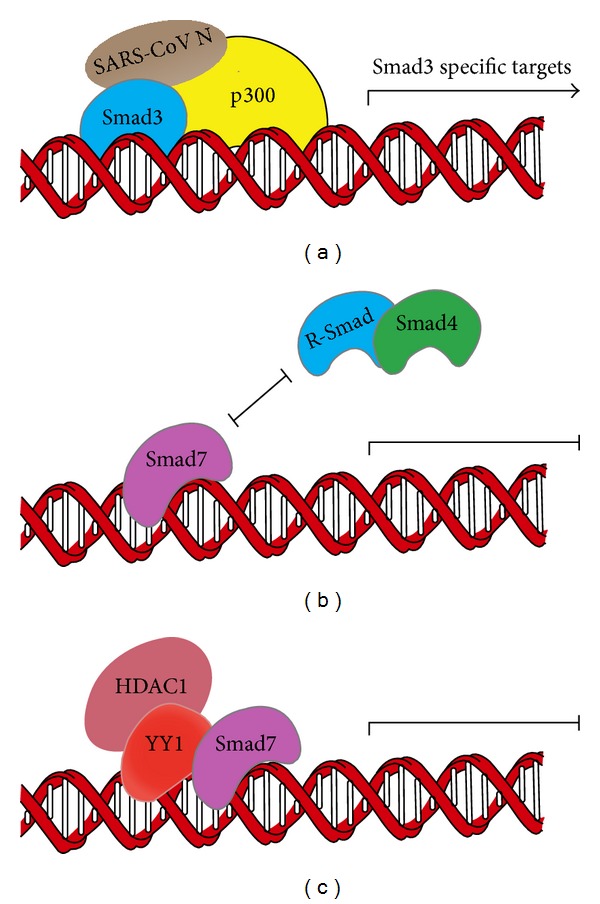
Regulation of Smad activity in the nucleus. (a) SARS-CoV N protein interacts with Smad3 and enhances the Smad3-p300 interaction, potentiating the Smad3-mediated transcription of fibrotic genes. SARS-CoV N protein can also interfere with the complex formation between Smad3 and Smad4, thereby inhibiting Smad4-mediated expression of apoptotic genes. (b) Smad7 directly binds to DNA and represses TGF-*β* signaling by interfering with the functional R-Smad/Smad4-DNA complex on target gene promoters. (c) YY1 can cooperate with Smad7 to inhibit TGF-*β* signaling in the nucleus via recruiting HDAC1.

Smad4 is the common Smad critical for both TGF-*β*/activin and BMP signaling. However, several studies have also revealed Smad4-independent R-Smad signaling [94–96]. Severe acute respiratory syndrome-associated coronavirus nucleocapsid protein (SARS-CoV N protein) is a 46 kDa viral RNA-binding protein that shares little homology with the N proteins of other known coronaviruses [97]. We found that SARS-CoV N interacts with Smad3 and enhances Smad3-p300 interaction, which specifically potentiates the Smad3-mediated transcriptional responses of TGF-*β* such as the expression of plasminogen activator inhibitor-1 (PAI-1) [98]. At the same time, the SARS-CoV N interferes with the complex formation between Smad3 and Smad4 and inhibits TGF-*β*-induced Smad4-mediated proapoptotic genes expression and cell apoptosis (Figure 4(a)).

In addition, we reported that in some cell lines, including Hep3B, HeLa, L17 cells (a mutant mink lung epithelial Mv1Lu cell line lacking T*β*RI) and human normal lung epithelial HPL-1 cells, Smad7 is predominantly localized in the nucleus and can inhibit the transcriptional activity of the functional R-Smad-Smad4 complex, independently, of inhibition of the type I receptors [99]. Unlike R-Smads and Smad4, which bind to DNA through their MH1 domains, biotinylated oligonucleotide pull-down assays and single-molecule force spectroscopy studies showed that Smad7 binds to DNA through its MH2 domain and thus represses TGF-*β* signaling by interfering with the functional R-Smads/Smad4-DNA complex formation on the target gene promoters [99, 100] (Figure 4(b)). These results suggest that Smad7 can inhibit TGF-*β* signaling in the nucleus by a novel mechanism.

Furthermore, we identified Yin Yang 1 (YY1), a ubiquitously expressed transcription repressor, as a critical cooperator of Smad7 in the nucleus [101]. Although it has been reported that YY1 can attenuate TGF-*β*/Smad signaling independently of its DNA binding ability [102], we found that YY1 and Smad7 could interact with each other and synergistically suppress TGF-*β*-induced transcription in the nucleus. Mechanistically, Smad7 enhances the interaction of YY1 with the histone deacetylase HDAC1 (Figure 4(c)). These studies reveal the important function of Smad7 to attenuate TGF-*β* signaling in the nucleus. This notion is supported by a recent report showing that nuclear Smad7 can promote myogenesis independent of TGF-*β*/Smad3 signaling [103].

## 7. Conclusions and Perspectives

Modulating the activity and stability of TGF-*β* receptors is a critical step for regulation of TGF-*β* signaling. Although much effort has been made to understand the regulatory mechanisms of TGF-*β* receptors, many important questions still remain unsolved. For instance, although degradation of TGF-*β* receptors is sensitive to the inhibitors of lysosome and proteasome, it is unclear how these two degradation pathways cooperate to achieve full degradation of TGF-*β* receptors. In addition to the caveosome pathway, TGF-*β* receptors can be transported to lysosomes via early endosomes and later endosomes. How is the intracellular sorting of TGF-*β* receptors regulated? Ubiquitination is known to promote TGF-*β* receptors degradation. However, its role in mediating TGF-*β* receptors partition and internalization is unclear. In addition, how the receptors in lipid rafts activate MAPK is another important subject of future investigation.

For Smad regulation, many questions await to be addressed too. It is well documented that the TGF-*β* receptor-mediated C-terminal phosphorylation of Smad2/3 is the key event for Smad activation. TGF-*β* receptors can also induce the Smad2/3 phosphorylation in the linker region [104, 105]. The linker phosphorylation has been shown to inhibit Smad activity or induce Smad degradation [6]. How the inhibitory linker phosphorylation and the activating C-terminal phosphorylation are coordinated is unknown. In the nucleus, Smad7 can bind to DNA via its MH2 domain and inhibit TGF-*β*-driven transcription by interfering with the R-Smad/Smad4-DNA association. It will be interesting to investigate whether Smad7 has other function independent of inhibition of TGF-*β* signaling.

Regulation of TGF-*β* signaling has been extensively investigated. However, as TGF-*β* signaling controls a wide range of biological responses and distinct regulatory mechanism is employed by different tissue at different time, exploration of the molecular mechanisms of how the TGF-*β* signaling is modulated in specific pathological or physiological processes will be an exciting field.
